# Directly targeting ASC by lonidamine alleviates inflammasome-driven diseases

**DOI:** 10.1186/s12974-022-02682-w

**Published:** 2022-12-28

**Authors:** Chen Chen, YuWei Zhou, XinPeng Ning, ShengLong Li, DongDong Xue, CaiLv Wei, Zhu Zhu, LongXiang Sheng, BingZheng Lu, Yuan Li, XiaoYuan Ye, YunZhao Fu, Chuan Bai, Wei Cai, YuXuan Ding, SuiZhen Lin, GuangMei Yan, YiJun Huang, Wei Yin

**Affiliations:** 1 Department of Pharmacology, Zhongshan School of Medicine, Sun Yat-sen University, Guangzhou, 510080 China; 2 Department of Molecular Biology and Biochemistry, Zhongshan School of Medicine, Sun Yat-sen University, Guangzhou, 510080 China; 3 State Key Laboratory of Ophthalmology, Zhongshan Ophthalmic Center, Sun Yat-sen University, Guangzhou, 510060 China; 4 Institute of Human Virology, Key Laboratory of Tropical Disease Control of Ministry of Education, Zhongshan School of Medicine, Sun Yat-sen University, Guangzhou, 510080 China; 5 Guangzhou Cellprotek Pharmaceutical Co., Ltd., Guangzhou, 510663 China

**Keywords:** ASC inhibitors, Inflammasome, Lonidamine, Multiple sclerosis, Ischemic stroke, Sepsis

## Abstract

**Background:**

Dysregulated activation of the inflammasome is involved in various human diseases including acute cerebral ischemia, multiple sclerosis and sepsis. Though many inflammasome inhibitors targeting NOD-like receptor protein 3 (NLRP3) have been designed and developed, none of the inhibitors are clinically available. Growing evidence suggests that targeting apoptosis-associated speck-like protein containing a CARD (ASC), the oligomerization of which is the key event for the assembly of inflammasome, may be another promising therapeutic strategy. Lonidamine (LND), a small-molecule inhibitor of glycolysis used as an antineoplastic drug, has been evidenced to have anti-inflammation effects. However, its anti-inflammatory mechanism is still largely unknown.

**Methods:**

Middle cerebral artery occlusion (MCAO), experimental autoimmune encephalomyelitis (EAE) and LPS-induced sepsis mice models were constructed to investigate the therapeutic and anti-inflammasome effects of LND. The inhibition of inflammasome activation and ASC oligomerization by LND was evaluated using western blot (WB), immunofluorescence (IF), quantitative polymerase chain reaction (qPCR) and enzyme-linked immunosorbent assay (ELISA) in murine bone marrow-derived macrophages (BMDMs). Direct binding of LND with ASC was assessed using molecular mock docking, surface plasmon resonance (SPR), and drug affinity responsive target stability (DARTS).

**Results:**

Here, we find that LND strongly attenuates the inflammatory injury in experimental models of inflammasome-associated diseases including autoimmune disease-multiple sclerosis (MS), ischemic stroke and sepsis. Moreover, LND blocks diverse types of inflammasome activation independent of its known targets including hexokinase 2 (HK2). We further reveal that LND directly binds to the inflammasome ligand ASC and inhibits its oligomerization.

**Conclusions:**

Taken together, our results identify LND as a broad-spectrum inflammasome inhibitor by directly targeting ASC, providing a novel candidate drug for the treatment of inflammasome-driven diseases in clinic.

**Supplementary Information:**

The online version contains supplementary material available at 10.1186/s12974-022-02682-w.

## Introduction

Inflammation is the immune system's response to pathogenic infections, tissue damage, and irritants, which is tightly regulated by the organism and usually beneficial to the host. However, when the inflammatory response becomes persistent or uncontrollable, it leads to increased damage and promotes immunopathology. Persistent inflammation has been recognized as a driver of several diseases, including neurological disorders such as Alzheimer’s disease [1], multiple sclerosis (MS) [2], stroke [3] and traumatic brain injury (TBI) [4], metabolic diseases [5, 6], tumors [7] and infectious diseases such as sepsis [8].

Inflammasomes are cytosolic multimolecular protein complexes which respond to innate immune recognition induced by microbial infections and endogenous danger signals [9, 10]. Inflammasomes are composed of a pattern recognition receptor (PRR), an adaptor protein called ASC and caspase-1. Several kinds of PRRs have been identified, including NOD (nucleotide-binding oligomerization domain), the NLR (leucine-rich repeat (LRR)-containing protein) family members like NLRP1, NLRP3 and NLRC4, and the proteins absent in melanoma 2 (AIM2) and pyrin [11]. Among them, NLRP3 is particularly important because it can be activated in response to a wide variety of stimuli, including bacteria, viruses, extracellular ATP, pollutants, metabolic dysregulation and tissue damage [10–12]. Once activated, the sensor binds to ASC and promotes its oligomerization to form a single macromolecular aggregate known as the ASC speck that activates caspase-1 [13, 14]. Active caspase-1 mediates the maturation and secretion of proinflammatory mediators, including IL-1β and IL-18 and also induces pyroptosis through the cleavage of gasdermin D (GSDMD) [15].

ASC oligomerization is required for the assembly of diverse inflammasomes [9]. In addition, extracellular ASC specks have been confirmed to have novel functions of inflammatory signals [16] to induce neutrophil infiltration [17], accelerate Aβ deposition [18] and regulate adaptive immunity [19]. Increased ASC specks were detected in serum of patients with chronic respiratory diseases or autoinflammatory diseases [17, 20], and in cerebrospinal fluid of patients with subarachnoid hemorrhage and TBI, acting as potential biomarkers of these diseases [17, 20, 21]. These findings strongly suggest that targeting ASC may be a new strategy for the treatment of inflammatory diseases.

Small-molecule inhibitors of inflammasomes have been designed and developed with distinct strategies. Some compounds directly block the activation of the NLRP3 inflammasome, such as MCC950 [22], CY-09 [23], OLT1177 [24], tranilast [25] and oridonin [26]. Other compounds, such as glyburide [27], BHB [28] and fenamate [29] can suppress the upstream signals of NLRP3 activation. However, due to the diverse biological processes of these upstream signals, these compounds are likely to produce off-target effects. Although these inhibitors have shown therapeutic efficacy against various experimental animal models of immunopathological diseases, there are no clinically available NLRP3 inhibitors. The highly selective and potent NLRP3 inhibitor MCC950 was tested, but was suspended in a phase II clinical trial for rheumatoid arthritis due to hepatotoxicity [30]. OLT1177 has entered phase II clinical trials for arthritis [31]. Whether these drugs can be used to treat NLRP3 inflammasome-related diseases in humans remains to be determined. Moreover, few inhibitors have been reported to suppress the inflammasomes types other than NLRP3 inflammasome.

Lonidamine (LND) is a derivate of indazole-3-carboxylic acid that can inhibit the rate-limiting glycolytic enzyme hexokinase 2 (HK2) [32]. LND is firstly reported as the candidate of a contraceptive agent, and then is applied as antineoplastic drug due to its anti-Warburg effect. The anti-inflammatory effect of LND was observed in animal models of ischemic stroke [33] and arthritis [34]. Moreover, LND significantly inhibited the IL-1β release in microglia [33] or THP1 [34] after the stimulation of hypoxia and LPS, respectively, indicating the potential role of LND on inflammasomes activation.

Here, we demonstrate that LND blocks inflammasome activation by directly targeting ASC in a HK2-independent manner and shows therapeutic effects in animal models of multiple inflammasome-related disease, including MS, ischemic stroke and sepsis. In conclusion, our study identifies LND as a novel inflammasome inhibitor via directly targeting ASC.

## Materials and methods

### Reagents and antibodies

Anti-IL-1β antibodies (AF-401-NA) were from RD Systems, anti-GSDMD (ab209845) and anti-NEK7 antibodies (ab133514) were from Abcam, anti-caspase-1 (AG-20B-0042B) and anti-ASC antibodies (AG-25B-0006) were from Adipogen, and the anti-αtubulin antibodies (ARG65693) were from Arigo. LPS (*Escherichia coli* O111:B4; #L2630), ATP (A6419), and nigericin (481990) were obtained from Sigma. LND (AF-1890) and imiquimod (R-837) were obtained from Selleck. MSU crystals (tlrl-msu), muramyldipeptide (tlrl-mdp), flagellin from *S. typhimurium* (tlrl-stfla) and poly(dA:dT)/LyoVec™ were from Invivogen. Anti-hexokinase 2 antibodies (NBP2-02272) were from Novus. Anti-NLRP3 antibodies (15101) were from Cell Signaling Technology. Goat anti-rabbit IgG (HRP) (arg65351), donkey anti-goat IgG (HRP) (arg65352) and goat anti-mouse IgG antibodies (HRP) (arg65350) were from Arigo. Disuccinimidyl suberate (DSS) was purchased from Thermo Scientific.

### Mice

C57BL/6J mice were obtained from Guangdong Medical Laboratory Animal Center (Guangzhou, China). Mice (B6.129P2(Cg)-Hk2^tm1.1Uku^/Kctt, HK2 CKO flox/flox) with conditional knockout of hexokinase II were introduced from the European Mouse Mutation Repository (EMMA, item number: 02074) and were bred by Guangdong Pharmachem Biotechnology Co. (B6J. B6n (Gg) -cx3cr1^tm1.1 (CRE) jung/j^, Cx3cr1-Cre T) mice are from Saiye (Guangzhou) Biotechnology Co., Ltd. All animal experiments in this study were approved by the Institutional Animal Care and Use Committee and the Laboratory Animal Ethics Committee of Sun Yat-sen University (No. SYSU-IACUC-2022-B0070).

### Cell preparation and stimulation

BMDMs were isolated from 6- to 8-week-old mouse bone marrow and cultured for 7 to 8 days in Dulbecco’s modified Eagle’s medium (DMEM) supplemented with 10% FBS and 20% supernatant from L929 cells (ATCC). CX3CR1-cre × HK2^flox/flox^ mice was used to selectively knockout HK2 in the monocyte-macrophage lineage, and then the primary BMDMs were isolated from these knockout mice. J774A.1 cells were obtained from ATCC and cultured in DMEM supplemented with 10% FBS.

To induce NLRP3 inflammasome activation, BMDMs (5 × 10^5^ cells/ml) and J774A.1 cells (3 × 10^5^ cells/ml) were plated in 6-well plates and incubated overnight, and the medium was changed to fresh DMEM the following morning. Then, the cells were primed with LPS (500 ng/mL) for 3 h. After that, LND was added to the culture and incubated for another 30 min, and then, the cells were stimulated with ATP (5 mM) for 30 min, MSU (150 μg/mL), imiquimod (100 μM), and nigericin (10 μM) for 4 h. Cell extracts and collected supernatants were analyzed by immunoblotting.

For NLRP1 inflammasome activation, BMDMs were primed with LPS (500 ng/mL) for 4 h before LND treatment for 30 min and subsequent muramyl dipeptide (MDP) (500 ng/mL) stimulation for 6 h. In the case of NLRC4 stimulation, after the LPS incubation for 4 h, cells were pretreated with LND for 30 min and then were stimulated for 8 h by Standard flagellin (1 μg/mL) from S. typhimurium (FLA-ST). To induce AIM2 inflammasome activation, cells were incubated with LPS for 4 h and then treated by LND for 30 min. After that the dsDNA (2 μg/mL) complexed with LyoVec was added to medium for 8 h.

### LPS-induced sepsis model

Eight-week-old male C57BL/6 mice were injected intraperitoneally with LND (40 mg/kg and 60 mg/kg) or vehicle control (β-cyclodextrin) 0.5 h before intraperitoneal injection of LPS (15 mg/kg) (Sigma–Aldrich, L2630). After 4 h, the mice were euthanized, and the serum levels of IL-1β and IL-18 were measured by ELISA kits according to the manufacturer’s instructions.

#### MCAO model

Ten-week-old male C57BL/6 mice underwent MCAO surgery as previously described [35]. Before the operation, the animals fasted overnight but were given drinking water. The next day, the mice were weighed and anesthetized with isoflurane. During the surgery, body temperature was maintained at 37 ± 0.5 °C using a heating pad. The unilateral common carotid artery and external carotid artery were then exposed and ligated. A 0.28-mm nylon monofilament with a silicone-coated tip was inserted into the internal carotid artery, thereby occluding the origins of the anterior cerebral and middle cerebral arteries. After 60 min of ischemia, reperfusion was accomplished by withdrawing the nylon monofilament. The mice were subjected to assays of Longa’s neurobehavioral scores (0–5 points). Mice rated 0 and 5 were excluded. Then, the mice were intraperitoneally injected with LND (60 mg/kg). All surgeries were performed under anesthesia. After the surgery, the animals were given agar gel. After 23 h of reperfusion, the neurobehavioral scores of the mice were determined according to the Clark scoring method [36]. Then, the mice were anesthetized with isoflurane and perfused with normal saline and paraformaldehyde, and the brain tissue was collected for Western blotting and paraffin embedding. All efforts were made to minimize animal suffering and to reduce the number of animals used.

### Induction and assessment of EAE

This modeling method is based on previous research [37]. Eight-week-old female C57BL/6 mice were injected subcutaneously in the dorsal flanks with 150 μg MOG_35-55_ peptide (RS Synthesis) emulsified in CFA (5 μg/mL). Pertussis toxin (500 ng per mouse, list labs) was given i.v. 24 h later. A second MOG and pertussis injection was given 7 d later. LND was intraperitoneally injected daily (60 mg/kg) beginning at disease induction. Mice in the vehicle group were injected with β-CD at the same time points. Animals were monitored and scored daily until they were euthanized. Clinical scores were assessed as follows: 0 = no symptoms, 1 = loss of tail tonicity, 2 = unsteady gait, 3 = hind limb paralysis, 4 = forelimb paralysis, 5 = moribund or dead. If scores fell between two points, 0.5 gradations were used.

### ASC oligomerization assay

BMDMs were plated in 6-well plates and washed with ice-cold PBS, and 500 µl of ice-cold buffer (20 mM HEPES–KOH, pH 7.5, 150 mM KCl 1% NP40, protease inhibitor) was added. Cells were lysed for 30 min at 4 °C, and 50 µL of the lysate was removed for Western blotting. Then, the remaining lysates were centrifuged at 2500 × *g* for 10 min at 4 °C. The pellets were resuspended in 500 µl of ice-cold PBS. Then, 2 mM disuccinimidyl suberate (Thermo Fisher A39267) was added to the resuspended pellets and incubated for 30 min with rotation at room temperature. The samples were then centrifuged at 2500 × *g* for 10 min at 4 °C. The supernatants were removed, and the crosslinked pellets were resuspended in 30 µl of Laemmli buffer. The samples were boiled for 10 min at 100 °C and analyzed by Western blotting.

### ELISA

Mouse IL-1β (LIANKE BIOTECH, EK201B/3), IL-18 (LIANKE BIOTECH, EK218) and TNF-α (LIANKE BIOTECH, EK282/3) in cell culture supernatants and serum were analyzed according to the manufacturer’s instructions.

### Immunoprecipitation (IP) and pulldown assay

LPS (500 ng/ml)-primed BMDMs were treated with LND for 30 min following stimulation with ATP for 30 min. After that, the cells were resuspended in IP lysis buffer (Beyotime, P0013J) with protease inhibitors. Then, the samples were centrifuged at 12,000 *g* at 4 °C for 10 min. The supernatants were incubated overnight at 4 °C with primary antibodies or normal rabbit/mouse IgG. The next day, the antibody–protein complexes were precipitated by protein G beads at 4 °C for 2 h and subjected to immunoblot analysis.

### Immunohistochemical staining

Brain and spinal cord samples were analyzed according to the manufacturer’s instructions of the immunohistochemistry (IHC) staining kit (Abcam, ab80436, Cambridge, MA, USA). Briefly, after deparaffinization and hydration, the slices were placed in 3% hydrogen peroxide for 15 min and subjected to antigen retrieval for 20 min, after which they were cooled to room temperature. Sections were stained with H&E, Nissl or with LFB, respectively. The samples were incubated with the indicated primary antibodies overnight at 4 °C in an antibody diluent with background reducing agents (DAKO, S3022, Santa Clara, CA, USA). Then, after being washed with PBS 3 times, the slices were sequentially stained with a diaminobenzidine (DAB) substrate–chromogen mixture and hematoxylin.

Images of the slices were captured using an inverted microscope (Eclipse Ti-U, Nikon, Japan). Image analysis was carried out blindly using Image-Pro Plus software (version 6.0, Media Cybernetics, Inc., Silver Springs, MD, USA).

### Immunofluorescence staining

During tissue sectioning, the brain and spinal cord were routinely separated and fixed. Then, the samples were embedded in paraffin and cut into 5-μm-thick sections. After deparaffinization and hydration, the slices were subjected to antigen retrieval for 20 min and cooled to room temperature. The samples were incubated with the indicated primary antibodies overnight at 4 °C in an antibody diluent with background reducing reagents (DAKO, S3022, Santa Clara, CA, USA). The samples were washed with PBS 3 times and incubated with indicated fluorescence-conjugated secondary antibodies (Molecular Probes, Thermo Fisher Scientific, Rockford, IL, USA) for 1 h. Then, the samples were washed and stained with Hoechst 33342 (5 µg/mL, Sigma–Aldrich, St. Louis, MO, USA) for 10 min. The film was sealed with a water-soluble sealing agent. The slices were mounted and imaged by using a Nikon A1 Spectral Confocal Microscope (Nikon, Japan).

### Real-time reverse transcription PCR

Total RNA was extracted from cells using TRIzol® reagent (Invitrogen, 15596), followed by reverse transcription using oligo (dT) and RevertAid Reverse Transcriptase (Thermo Fisher Scientific, EP0442). Real-time PCR was performed with SuperReal PreMix SYBR Green (Tiangen, FP205) with a 7500 Fast Real-Time PCR System (Applied Biosystems, USA). The relative mRNA expression level was calculated and analyzed by the comparative Ct method (RQ = 2^−ΔΔCt^). The PCR primers for HK1, HK2, HK3, SDHA, SDHB, SDHC, SDHD, VDAC1, VDAC2, VDAC3, MPC1, MPC2, IL-1β, IL-18, TNF-α and β-actin synthesized by Invitrogen and the primer sequences were shown in Additional file 2.

### Flow cytometry

To analyze infiltrating immune cells in the CNS, brains tissue and spinal cords from MOG35–55-immunized mice were ground with a tissue homogenization instrument to form a single-cell suspension that was filtered through a 300-mesh filter membrane. After centrifugation, the single cell suspension was resuspended with 37% percoll and centrifuged for 30 min at 1000 × *g*, room temperature with the lowest acceleration settings and without brake. Mononuclear cells were isolated from the lowest layer. The cells were suspended in PBS containing 1% FBS. The cells were washed three times and stained with cell surface marker antibodies for flow cytometric analysis. The following antibodies were used: CD45-BV510 (BD, 563891), CD4-FITC (BioLegend, 100406), CD8-Alexa Fluor 700 (BD, 557959), and CD11b-BV421 (BioLegend, 101236). Flow cytometry analysis was performed by flow cytometry (CytoFLEX, Beckman Coulter). Cell debris and dead cells were excluded on the basis of forward and side scatter and Fixable Viability Stain 780 (BD, 565388).

### Cell death assays

BMDMs (5 × 10^5^ cells/ml) and J774A.1 cells (3 × 10^5^ cells/ml) were plated in 6-well plates and stimulated with LPS for 4 h, followed by treatment with various doses of LND for 30 min. After treatment with 5 mM ATP for 30 min, the medium was collected, and LDH release was assessed using the CytoTox96 nonradioactive cytotoxicity assay (Promega, G1780) according to the manufacturer’s instructions.

### siRNA-mediated gene silencing in BMDMs

RNA interference was conducted with Lipofectamine™ RNAiMAX (Invitrogen, 13778) according to the manufacturer’s instructions. Cells that achieved 60–70% confluence were subjected to RNA interference. Samples were collected 48 h after RNA interference and subjected to Western blot or real-time PCR as described above. Small interfering RNAs targeting HK1 and HK2 were synthesized by Guangzhou Ribo Biotechnology Co., Ltd., and the siRNA sequences were shown in Additional file 2.

### DARTS assay

DARTS was performed according to a published protocol [38]. BMDMs (5 × 10^5^ cells/ml) were plated in 10 cm dishes and incubated overnight, and the medium was changed to fresh DMEM the following morning. Then, the cells were primed with LPS for 4 h and lysed with M-PER (Thermo, 78501) lysis buffer. Lysates were cleared by centrifugation at 12,000 × *g* for 10 min at 4 °C, and a BCA protein concentration assay was performed to determine the protein concentration. The lysates were incubated with LND at 4 °C overnight. 80 μg of protein lysate were used per reaction. Then, 20 ng of pronase (Sigma, PRON-RO) per μg of protein was added and incubated for 30 min at room temperature. The digestion reaction was stopped by adding 20 × protease inhibitor cocktail and incubating the samples on ice for 10 min. Next, 5 × SDS-PAGE loading buffer was added to the samples to achieve a final concentration of 1 × SDS–PAGE loading buffer. Protein samples were analyzed by immunoblotting.

### Molecular simulation docking with LigandScout

Structure of full-length human ASC (PDB code 2KN6) was downloaded from PDB database and subjected to ligandscout 4.4.7 software. LND was docked into the CARD domain of ASC using the default settings. Molecular graphics was prepared by PyMOL.

### Surface plasmon resonance (SPR) analysis

The experiment was performed in Biacore T100. Recombinant human ASC and NLRP3 protein (Zeye Biotechnology) was immobilized to a CM5 sensor chip (BR100530, Cytiva). LND was dissolved in basic running buffer (PBS containing 1% DMSO) and injected into the flow cell at different concentrations with a flow rate of 5 μl/min at 25 °C. The sensor chip was washed with basic running buffer between each concentration. Proteins were immobilized in different channels on the same chip and the response values obtained by injecting blank basic running buffer in the same way were used as control. The kinetic parameters of the interaction and the affinity constants were calculated with Biacore T100 evaluation software.

### Statistics analysis

The data were analyzed by GraphPad Prism 8.0 software and are presented as the mean ± SEM. The statistics were analyzed by using an unpaired *t* test for two groups and multiple *t* test or one-way ANOVA with Tukey’s test for multiple groups. *, *P* < 0.05, **, *P* < 0.01, ***, *P* < 0.001, and ****, *P* < 0.0001 were considered statistically significant.

## Results

### LND alleviates inflammatory injury in ischemic stroke and EAE

**Fig. 1:**
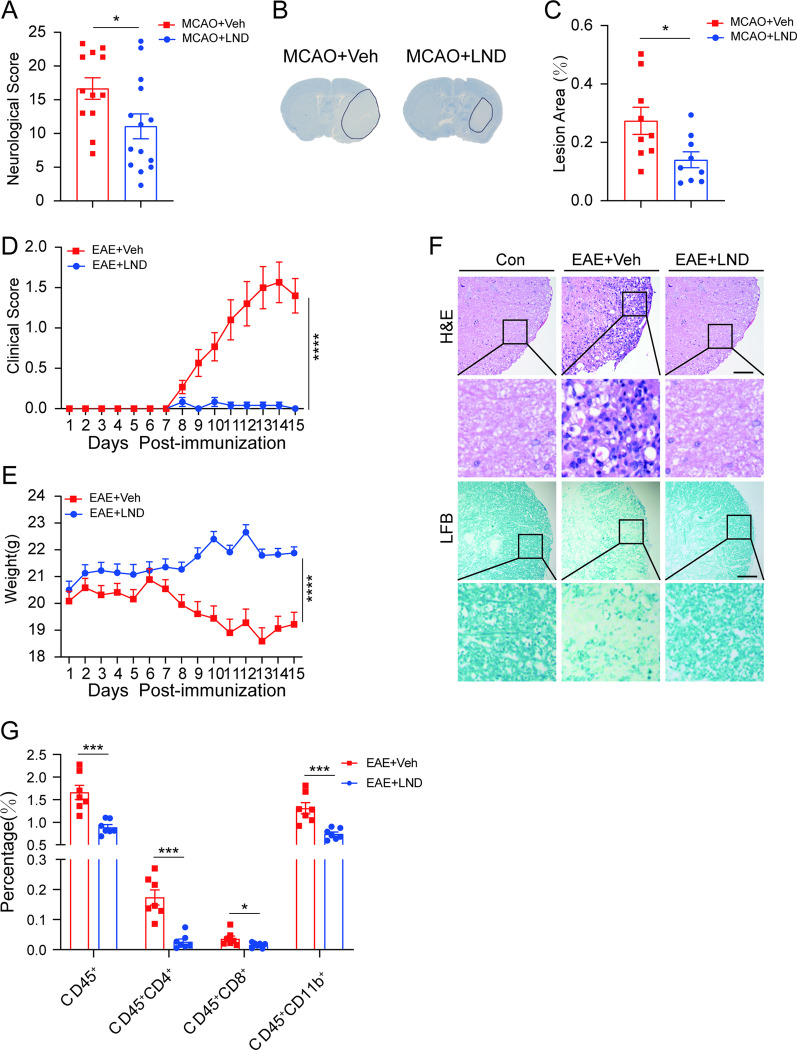
LND alleviates inflammatory injuries in experimental ischemic stroke and EAE. **A** The mice were subjected to MCAO and sham surgery for 1 h followed by intraperitoneal injection of LND (60 mg/kg) and 23 h of reperfusion. Behavioral scores after 23 h of reperfusion; *n* = 15. **B** and **C** Representative Nissl staining of brain sections (**B**) and quantification of the area of neuronal damage (**C**). *n* = 9. **D** EAE was induced in mice by MOG35 ~ 55 peptides and pertussis toxin as described in the Materials and methods. Clinical scores of mice administered vehicle or LND daily after EAE induction; *n* = 12. **E** Weight changes of mice after EAE induction; *n* = 12. **F** Representative H&E and Luxol Fast Blue (LFB) staining. Scale bar = 100 μm. **G** Percentage of CD45^+^ gated on live cells, and CD45^+^CD4^+^, CD45^+^CD8^+^ and CD45^+^CD11b^+^ cells gated on live cells from the mononuclear cell population in the CNS. The cells were isolated on day 15 after EAE induction. The data are expressed as the mean ± SEM *P < 0.05; **P < 0.01; ***P < 0.001; ****P < 0.0001; NS, not significant. Unpaired *t* test was used for A, C, D and E, and multiple unpaired *t* test for G

Stroke and MS have extensively proven to be inflammation-associated diseases. Stroke is the second leading cause of death worldwide and there are no effective neuroprotectants at present [39, 40]. Among the mechanisms that promote stroke pathogenesis, the microglia-mediated inflammatory response is of paramount importance. We previously reported that LND inhibited microglial activation [33]; here we further evaluated the efficacy of LND using MCAO models of acute ischemic stroke. We found that 1 h after occlusion, LND administration significantly improved neurological outcomes (Fig. 1A) and reduced the infarct size (Fig. 1B and 1C) induced by MCAO surgery.

EAE shares the neuropathological features of MS, which is an incurable and progressive chronic inflammatory disease of the central nervous system (CNS). We further evaluated the anti-inflammatory effect of LND using an EAE model previously reported [37]. Briefly, mice were pretreated with LND from the date of the first immunization with myelin oligodendrocyte glycoprotein (MOG)_35~55_ peptides to the endpoint of the experiment. We found that LND significantly ameliorated neurological deficits (Fig. 1D) and inhibited weight loss (Fig. 1E) at the peak of the disease. Moreover, histological analysis showed that LND reduced the infiltration of monocytes in the spinal cord and protected against the demyelination of neurons (Fig. 1F). To further determine whether LND could reduce the massive infiltration of peripheral immune cells into the CNS, which is one of the main pathological characteristics of EAE, the proportion of immune cells in the spinal cord and brain tissue from each group were analyzed by flow cytometry analysis. The results showed that mice treated with LND had significantly reduced infiltration of CD4^+^ T cells, CD8^+^ T cells and myeloid cells (CD45^+^ CD11b^+^) in the CNS (Fig. 1G). To further evaluate the therapeutic effect of LND on EAE, mice were given LND after the onset of the disease. Consistent with the above observations, LND significantly rescued the severity of EAE (Additional file 1: Fig. S1A) as well as weight loss (Additional file 1: Fig. S1B). Similarly, the infiltration of CD4^+^ and CD8^+^ T cells in the CNS of mice receiving LND was remarkably reduced, and the proportion of myeloid cells was reduced but with no statistical significance (Additional file 1: Fig. S1C).

Overall, LND treatment markedly attenuated inflammatory injuries in ischemic stroke and EAE, suggesting that LND is a potent inhibitor of inflammation.

### LND inhibits inflammasome activation in vivo*.*

The inflammasome is the major sensor of sterile inflammatory signals and is therefore a key trigger of inflammatory responses. A series of cellular and molecular events including hypoxia, released glutamate [41], increased ROS level [42, 43], mitochondrial dysfunction [44], ion imbalance [45], neuronal demyelination and death trigger the activation of inflammasomes [46–48], leading to subsequent irreversible inflammatory damages of stroke and multiple sclerosis. We previously showed that LND decreased the transcription of IL-1β in microglia [33], which is one of the substrates of the inflammasome. Here, we further examined the possibility for LND to inhibit inflammasome activation in vivo.

**Fig. 2:**
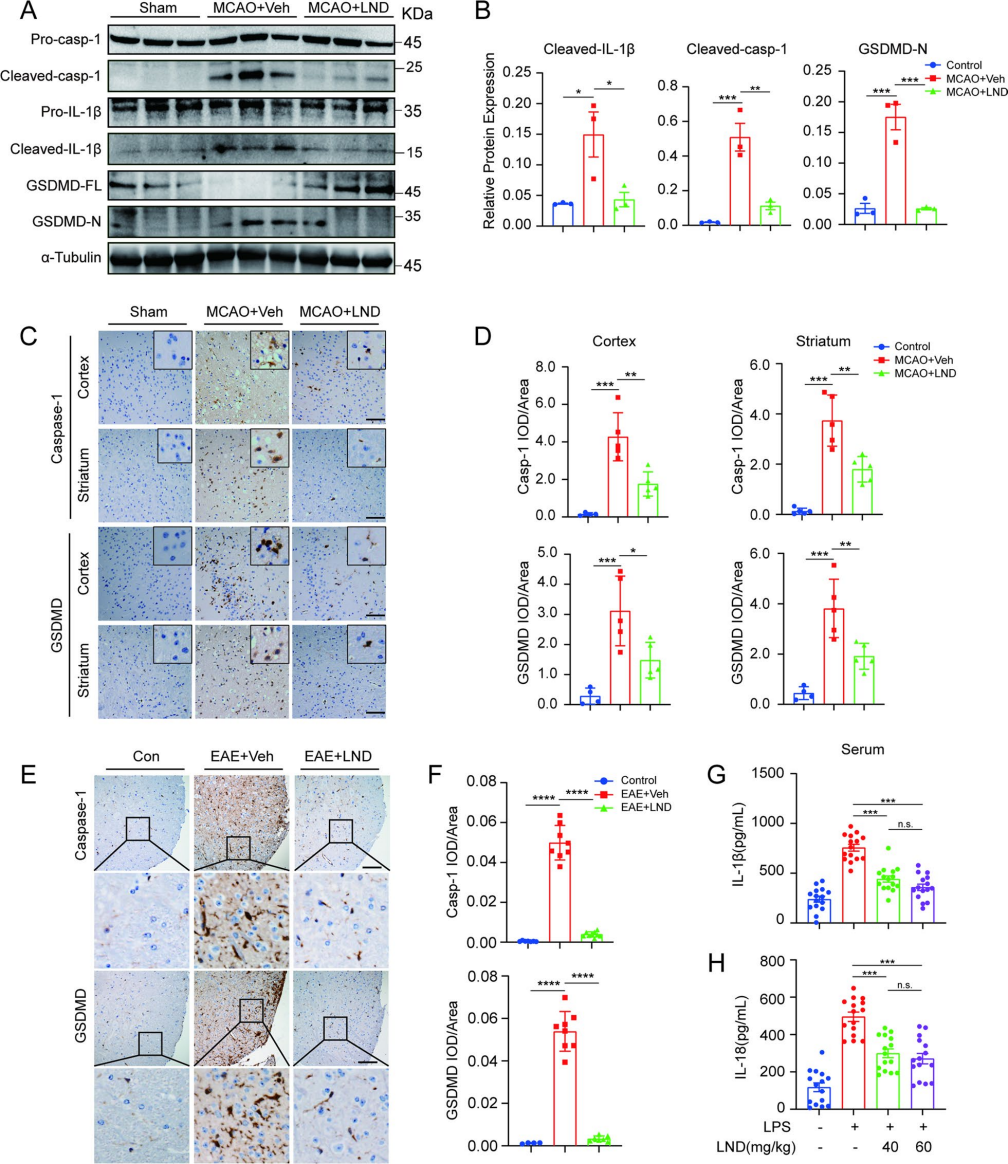
LND inhibits inflammasome activation in vivo. **A** and **B** Immunoblot (**A**) and quantification of gray values (**B**) of GSDMD, cleaved caspase-1 and cleaved IL-1β in brain tissue at 23 h of reperfusion after MCAO *n* = 3. **C** and **D** Immunohistochemical staining (**C**) and quantitative analysis (**D**) of GSDMD and caspase-1 at 23 h of reperfusion after MCAO in the cortex and striatum of the ischemic area. *n* = 5. Scale bar = 100 μm. **E** and **F** Immunohistochemical staining (**E**) and quantitative analysis (**F**) of GSDMD and caspase-1 in spinal cord tissue after EAE induction. *n* = 7. Scale bar = 100 μm. **G** and **H** ELISA analysis of IL-1β (**G**) and IL-18 (**H**) in the serum of mice that were intraperitoneally injected with LPS (15 mg/kg) for 4 h with or without pretreatment with LND. *n* = 15. The data are expressed as the mean ± SEM. *P < 0.05; **P < 0.01; ***P < 0.001; ****P < 0.0001; NS, not significant. The data were analyzed by one-way ANOVA and Tukey’s test

We found that LND treatment significantly abolished the increased protein levels of inflammasome products including cleaved IL-1β, cleaved caspase-1 and GSDMD-N in the infarcted brain tissues of MCAO mice (Fig. 2A and B). Immunohistochemical assay further indicated that LND treatment markedly reduced caspase-1 and GSDMD staining in the ischemic penumbra of the cortex and striatum of MCAO mice (Fig. 2C and D). Moreover, the significantly increased levels of GSDMD and caspase-1 in the spinal cords of EAE mice were markedly decreased by LND treatment (Fig. 2E and F). These results indicated that LND blocked inflammasome activation in CNS tissues of MCAO and EAE mice.

In addition to CNS inflammatory diseases, we further examined LND-mediated inhibition of the inflammasome in LPS-induced sepsis. Intraperitoneal injection of LPS could induce NLRP3-dependent inflammation and septic shock in mice [49]. As shown in Fig. 2G and H, LND treatment significantly alleviated the enhanced serum levels of IL-1β and IL-18 induced by LPS. All results above strongly suggested that LND inhibited inflammasome activation in vivo.

### LND suppresses NLRP3 inflammasome activation induced by various agonists

**Fig. 3:**
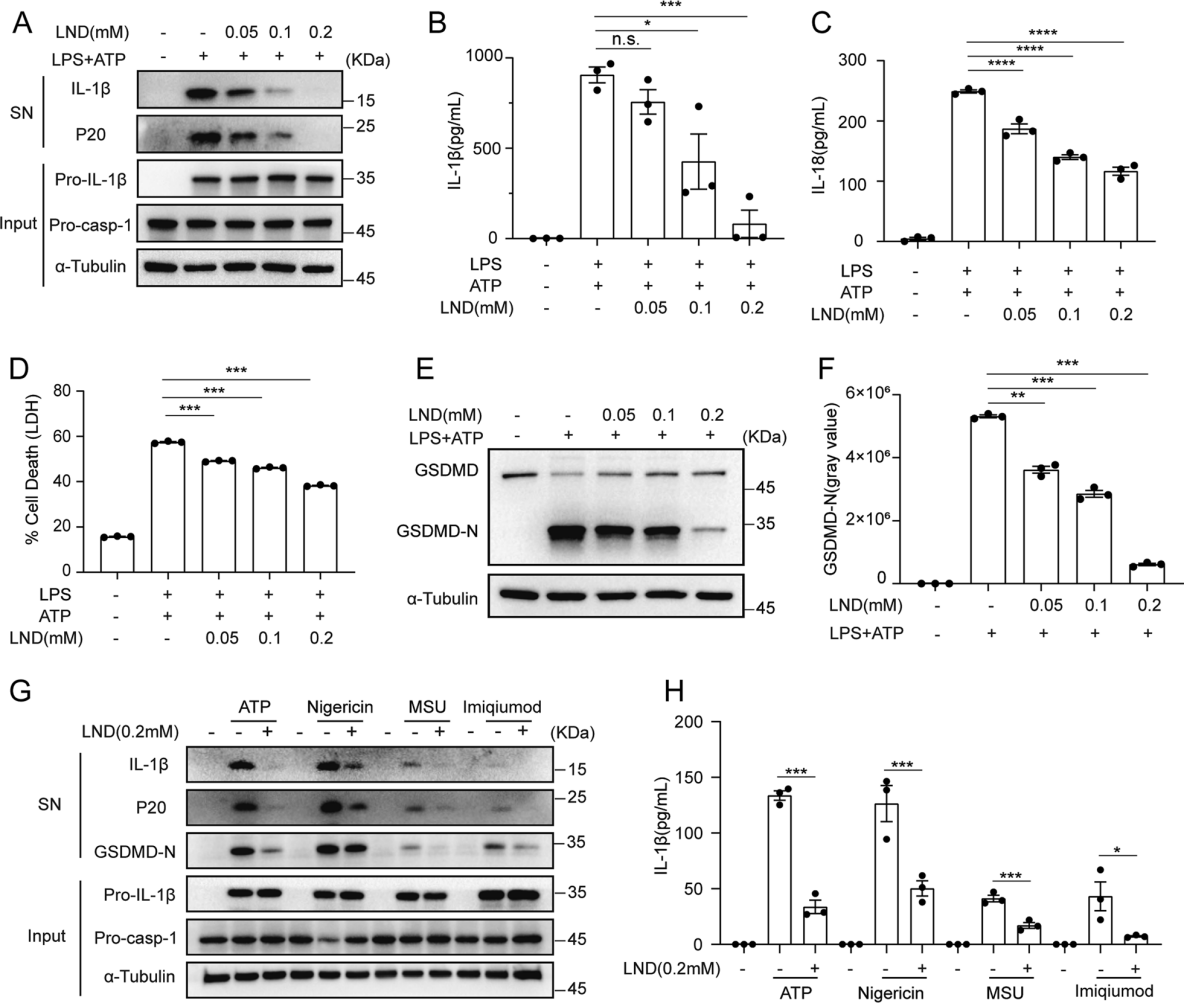
LND is a broad-spectrum inhibitor of the NLRP3 inflammasome. **A** Western blot analysis of mature IL-1β and cleaved caspase-1 (p20) in the supernatants (SN) of LPS-primed BMDMs treated for 0.5 h with various doses of LND and then stimulated with ATP (5 mM) for 0.5 h. Western blot analysis of pro-IL-1β and pro-caspase-1 in the lysates of BMDMs. **B** and **C** ELISA analysis of IL-1β (**B**) and IL-18 (**C**) in the supernatants of BMDMs. **D** LDH release in the supernatants of BMDMs. **E** Western blot analysis of GSDMD. **F** Quantitative analysis of the gray value of GSDMD-N. **G** and **H** LPS-primed BMDMs were pretreated with or without LND (200 μM) for 30 min and were then stimulated with nigericin, ATP, MSU or imiquimod. Western blot analysis of mature IL-1β and cleaved caspase-1 (p20) in the SN and of pro-IL-1β and pro-caspase-1 in the lysates of BMDMs (**G**). ELISA analysis of IL-1β (**H**) in the supernatants of BMDMs. The data are expressed as the mean ± SEM. *P < 0.05; **P < 0.01; ***P < 0.001; ****P < 0.0001; *NS*, not significant. The data were analyzed by one-way ANOVA and Tukey’s test

Using a classic NLRP3 inflammasome activation induced by the combined treatment of LPS and ATP in BMDMs, LPS-primed BMDMs were pretreated with LND for 0.5 h before being exposed to ATP to confirm the inhibitory effect of LND on inflammasome activation. Immunoblotting results showed that LND dose-dependently suppressed ATP-induced cleavage of pro-caspase-1 into P20 and the processing of the biologically active P17 form of IL-1β in BMDMs (Fig. 3A) and J1774A.1 cells (Additional file 1: Fig. S2A). Moreover, ELISA assay also indicated that LND inhibited the secretion of IL-1β (Fig. 3B) and IL-18 (Fig. 3C) in BMDMs and J1774A.1 cells (Additional file 1: Fig. S2B), but had no effect on inflammasome-independent production of the cytokine TNF-α (Additional file 1: Fig. S2C). LND also inhibited the subsequent LDH release (Fig. 3D and Additional file 1: Fig. S2D) and cleavage of GSDMD, the pyroptosis executioner (Fig. 3E and F). These results indicate that LND can effectively inhibit NLRP3 inflammasome activation and pyroptosis. We then examined whether LND affected the LPS-induced priming stage of inflammasome activation. When BMDMs were pretreated with LND before 3 h of LPS challenge, LND neither affected enhanced mRNA levels of pro-IL-1β, NLRP3, pro-IL-18 or TNF-α (Additional file 1: Fig. S2E–G), nor inhibit increased protein abundances of pro-IL-1β and NLRP3 (Additional file 1: Fig. S2H), indicating that LND had no effect on the priming phase of inflammasome.

In addition to ATP, the NLRP3 inflammasome can also be activated by other danger-associated molecular patterns, such as nigericin and monosodium urate (MSU) that depend on cellular potassium efflux [50–53]. Treatment with LND inhibited caspase-1 cleavage and IL-1β secretion triggered by nigericin and MSU (Fig. 3G and H). Moreover, LND also inhibited activation of caspase-1 and IL-1β induced by imiquimod (Fig. 3G and H) which is a potassium efflux-independent NLRP3 inflammasome activator [54]. Taken together, these results indicate that LND can inhibit NLRP3 inflammasome activation induced by various agonists in vitro*.*

### LND inhibits NLRP3 inflammasome activation independent of HK2

**Fig. 4:**
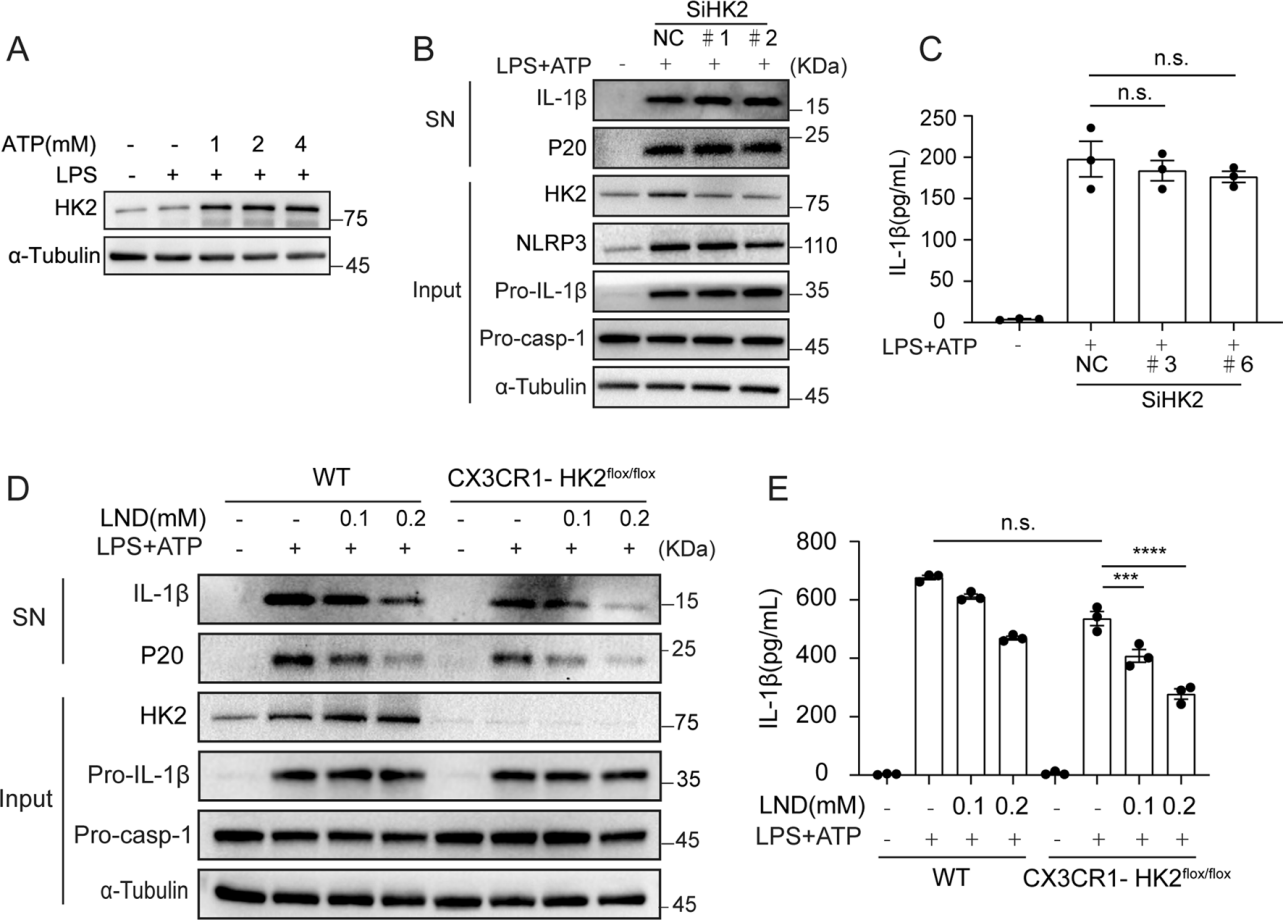
LND inhibits NLRP3 inflammasome activation independent of HK2. **A** Western blot analysis of HK2 expression in LPS-primed BMDMs treated with various doses of ATP for 30 min. **B** Western blot analysis of IL-1β and cleaved caspase-1 (p20) in the supernatants (SN) and pro-IL-1β, pro-caspase-1 and HK2 in the lysates of LPS-primed BMDMs transfected with control, scrambled siRNA or HK2-specific siRNA as indicated for 24 h and then stimulated with ATP. **C** ELISA analysis of IL-1β in the supernatants of BMDMs. **D** Western blot analysis of IL-1β and cleaved caspase-1 (p20) in the culture supernatants (SN) of LPS-primed BMDMs from HK2^−/−^mice treated for 30 min with LND and then stimulated with ATP. Western blot analysis of pro-IL-1β, pro-caspase-1 and HK2 in the lysates of those cells. **E** ELISA analysis of IL-1β in the supernatants of BMDMs. The data are expressed as the mean ± SEM *P < 0.05; **P < 0.01; ***P < 0.001; ****P < 0.0001; *NS*, not significant. The data were analyzed by one-way ANOVA and Tukey’s test

LND has been reported to suppress glycolysis by inhibiting the activity of HK2, and other pharmacological targets of LND, including voltage-dependent anion channel (VDAC), the mitochondrial pyruvate carrier (MPC), monocarboxylate transporters (MCT) and succinate dehydrogenase (SDH) has been identified [32]. To examine the possibility for LND acting on its known targets to inhibit NLRP3 inflammasome activation, we tested the mRNA levels of these targets during NLRP3 activation induced by LPS and ATP in BMDMs. We found that only the mRNA levels of HK2 increased while other targets of LND did not change (Additional file 1: Fig. S3A). Immunoblot assays also confirmed that LPS and ATP treatment induced upregulation of HK2 protein level (Fig. 4A), suggesting a possible role of HK2 in NLRP3 inflammasome activation.

Unexpectedly, siRNA-mediated interference with HK2 did not reduce the cleavage of caspase-1 or IL-1β production in BMDMs (Fig. 4B and C) and J1774A.1 cells (Additional file 1: Fig. S3B and S3C). Moreover, NLRP3 inflammasome activation in BMDMs isolated from HK2-knockout mice was comparable to that in BMDMs from wild-type (WT) mice (Additional file 1: Fig. S3D), and LND still attenuated NLRP3 activation in BMDMs from HK2-knockout mice (Fig. 4D and E). Our results here suggested that LND inhibited NLRP3 activation in an HK2-independent manner.

### LND selectively blocks ASC oligomerization

We then further explored the possible mechanism by which LND inhibits NLRP3 inflammasome activation. A series of upstream events triggered NLRP3 inflammasome activation, including potassium efflux and ROS production [53, 55]. LND can inhibit NLRP3 inflammasome induced by diversity activators that are dependent on or independent of potassium ion efflux (Fig. 3). Furthermore, LND did not inhibit the production of ROS (Additional file 1: Fig. S4A). These results strongly suggested that LND does not act on these upstream events of NLRP3 inflammasome activation.

**Fig. 5:**
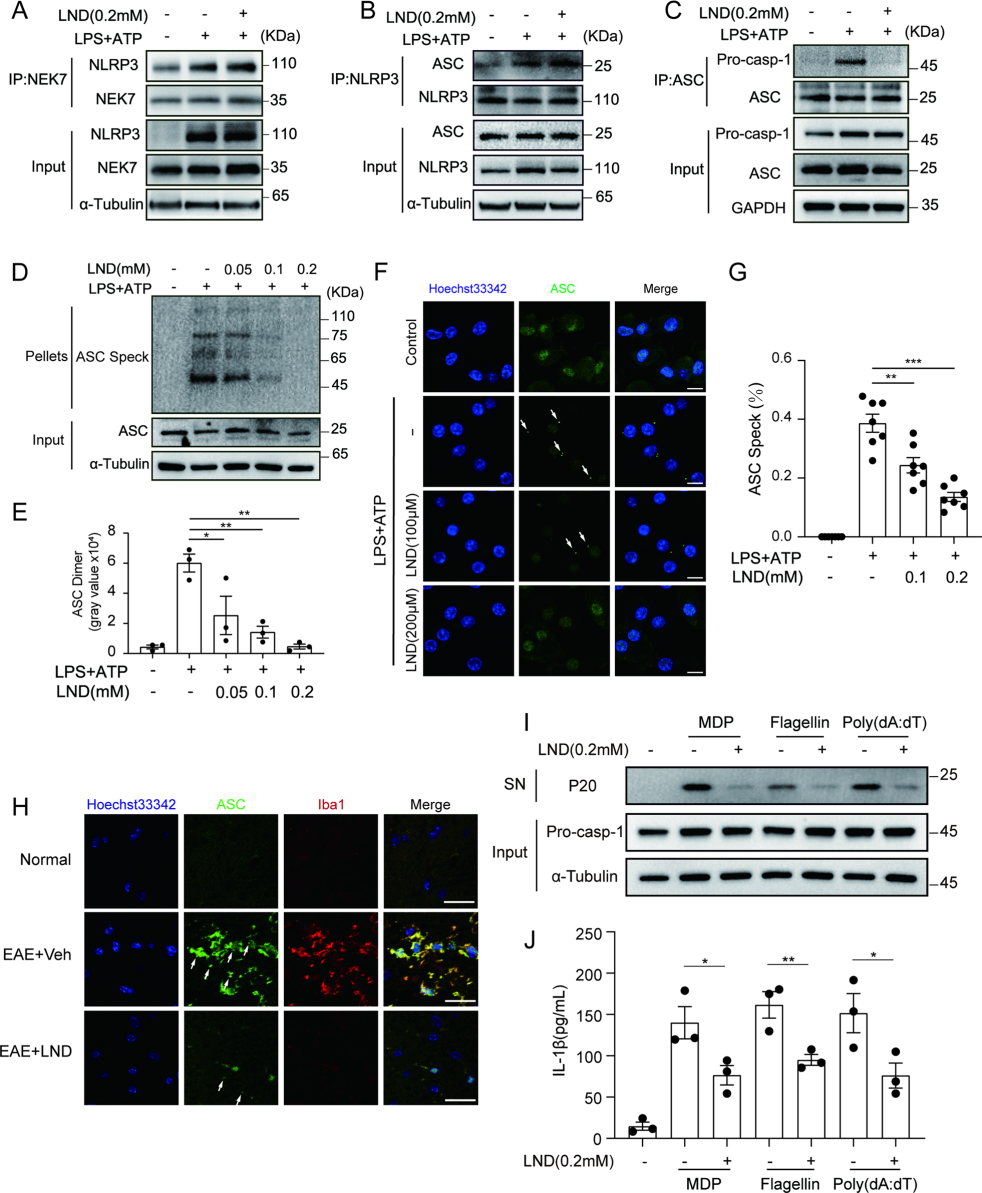
LND blocks the formation of ASC specks. **A**–**C** LPS-primed BMDMs were treated with LND (0.2 mM) for 30 min before 30 min of ATP treatment. Western blot analysis of NLRP3 protein in the cell lysates that immunoprecipitated with anti-NEK7 antibodies to evaluate the NLRP3-NEK7 interaction (**A**). Western blot analysis of ASC protein in the cell lysates that immunoprecipitated with anti-NLRP3 antibodies to evaluate the ASC-NLRP3 interaction (**B**). Western blot analysis of caspase-1 protein in the cell lysates that immunoprecipitated with anti-ASC antibodies to evaluate the pro-cas-1-ASC interaction (**C**). **D** and **E** LPS-primed BMDMs were treated with LND (0.2 mM) for 30 min before 30 min of ATP treatment. Western blot (**D**) and quantitative analysis (**E**) of crosslinked ASC in the NP-40–insoluble pellet with anti-ASC antibodies. **F** Immunofluorescence analysis of ASC speck formation in LPS-primed BMDMs treated with various doses of LND. Arrowheads depict ASC specks. Scale bar = 10 μm (**G**) The percentage of ASC speck in (**F**). **H** Immunofluorescence analysis of ASC in the spinal cords of EAE mice. White arrows depict ASC specks. Scale bar = 25 μm. **I** and **J** LPS-primed BMDMs were treated for 0.5 h with LND (0.2 mM) and then stimulated with inflammasome activators (ATP (5 mM, 30 min), poly(dA:dT) (1 μg/ml, 8 h), MDP (200 ng/ml, 8 h) or flagellin (200 ng/ml, 8 h)), respectively. Western blot analysis of cleaved caspase-1 (p20) in the supernatants (SN) of BMDMs and pro-caspase-1 in the lysates (**I**). ELISA analysis of IL-1β in the supernatants of BMDMs (**J**). The data are expressed as the mean ± SEM. *P < 0.05; **P < 0.01; ***P < 0.001; ****P < 0.0001; NS, not significant. The data were analyzed by one-way ANOVA and Tukey’s test

Then we try to figure out the possible role of LND directly acting on inflammasome assembly. Recently, NIMA-related kinase 7 (NEK7) has been recognized as an important component of the NLRP3 inflammasome, and NEK7-NLRP3 interactions are evidenced to be pivotal for NLRP3 oligomerization and inflammasome assembly [56]. However, LND treatment did not affect the interaction between NEK7 and NLRP3 in BMDMs (Fig. 5A). ASC-mediated connection of NLRP3 and caspase-1 is another key step for inflammasome assembly. We found that LND did not block the interaction between NLRP3 and ASC (Fig. 5B) but inhibited the binding between ASC and caspase-1 (Fig. 5C). Then we further examined the influence of LND on the oligomerization of ASC, which is a necessary step for inflammasome activation, and found that LND suppressed ATP-induced ASC oligomerization dose-dependently (Fig. 5D and E). Furthermore, ASC oligomerized formed a large speck in the perinuclear during the activation of inflammasomes in BMDMs (Fig. 5F and G), and pretreatment with LND remarkably inhibited this ASC speck formation (Fig. 5F and G). We also showed that the microglial ASC specks were strongly agitated in the spinal cords of EAE mice but could be alleviated by LND treatment (Fig. 5H).

In addition to NLRP3 inflammasome, ASC oligomerization has been reported to be required for activation of other types of inflammasome which mediated by receptors like AIM2, NLRP1 and NLRC4 [57–59]. Therefore, we also tested whether LND could block these inflammasomes activation other than NLRP3 inflammasome. Notably, LND inhibited the cleavage of caspase-1 (Fig. 5I) and the secretion of IL-1β (Fig. 5J) induced by MDP (an NLRP1 inflammasome activator), flagellin (an NLRC4 inflammasome activator) or poly(dA:dT) (an AIM2 inflammasome activator), respectively. Taken together, these results confirmed that LND could block ASC oligomerization, suggesting the possibility of direct binding for LND to ASC.

### LND directly binds to ASC

**Fig. 6:**
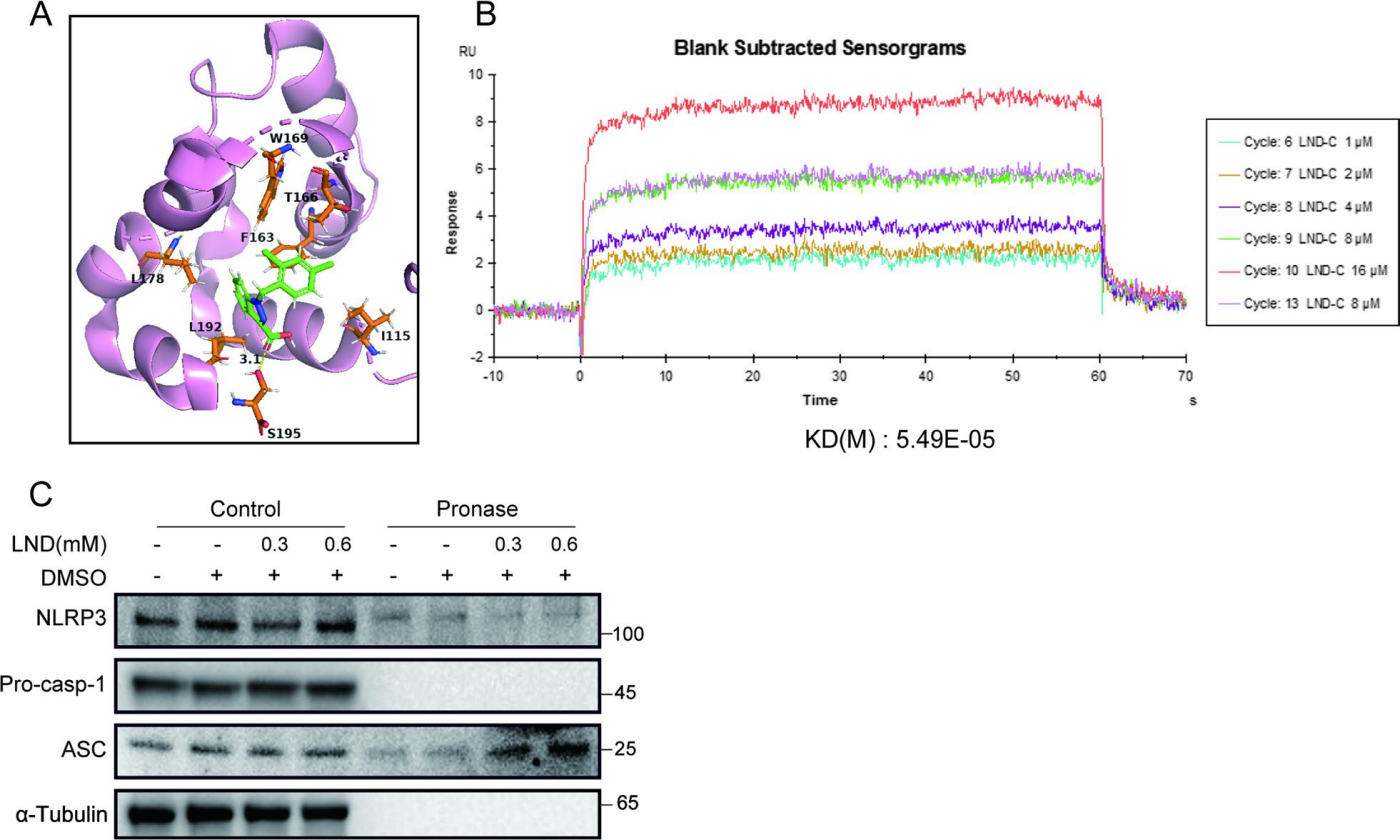
LND directly binds to ASC to inhibit NLRP3 assembly.** A** The docking model of ASC with LND using ligand scout software. LND is shown as a stick and colored green. The CARD domain of ASC is shown in cartoon and colored pink, while the interaction sites are colored orange. Yellow lines represent hydrogen bonds. **B** Sensorgrams showing LND binds to recombinant ASC protein were obtained from SPR analysis using Biacore. Different concentrations of LND are presented as an overlay plot aligned at the start of the injection. **C** LPS-primed BMDMs were lysed and incubated with LND overnight at 4 °C. DARTS assays were performed with pronase (20 ng/μg protein), and lysates were analyzed by immunoblotting

To investigate whether LND directly binds to ASC, we performed molecular modeling analysis using the crystal structure of ASC (PDB 2KN6). The results showed that LND readily docked into the CARD pocket and interacted with multiple loci including I155, F163, T166, W169, L178, L192 and S195 (Fig. 6A). Among them, W169, L178 and L192 have been reported to be critical for the oligomerization of ASC [58, 60]. Therefore, LND may block ASC oligomerization and ASC recruitment of pro-caspase-1 by interacting with these amino acids. Using a SPR assay to check the direct interaction of LND and recombinant human ASC protein, we found LND directly bound to ASC in a dose-dependent manner (Fig. 6B). While using the same batch of LND, our results also showed that LND did not interact with NLRP3 (Additional file 1: Fig. S5B), in contrast to the strong binding observed between LND and ASC (Additional file 1: Fig. S5A).We then used a DARTS approach based on drug binding stabilization of the target protein [38, 61] to confirm the interaction between LND and ASC. We found that LND protected ASC but not other components like NLRP3 and pro-caspase-1 from pronase-mediated protein degradation (Fig. 6C). These results reveal that LND selectively and directly binds to ASC.

## Discussion

Currently, many small-molecule inhibitors of NLRP3, most of which act by directly targeting NLRP3, are designed and develop to treat inflammatory diseases. However, there are still no clinically available inflammasome inhibitors [30]. As an antineoplastic drug, the safety of LND has been demonstrated in hundreds of patients in phase 3 clinical trials of breast and lung cancer, although with limited outcomes [62]. Recently, LND is further tested in clinical trials in combination with various chemotherapeutic agents [63, 64]. The anti-inflammatory effects of LND have been observed by our previous report in ischemic stroke-induced microglial activation [33] and LND attenuated the onset and severity of arthritis [34]. Our finding here further explores the novel role of LND on anti-inflammation through effective inhibiting NLRP3 inflammasome. Moreover, LND significantly alleviates the inflammatory injury of stroke, autoimmune encephalitis and LPS-induced sepsis, suggesting the possible clinical application of LND in inflammatory diseases.

ASC oligomerization and formation of ASC specks play a key role in inflammasome assembly. NLRP3 and AIM2, the two inflammasome receptors without CARD domains, require mediation of ASC for the activation of pro-caspase-1. While CARD-containing receptors such as NLRP1 and NLRC4, can directly recruit pro-caspase-1 [9]. However, ASC specks are also observed after NLRP1 and NLRC4 activation [57–59]. Our results reveal that LND directly targets ASC, which is required for the activation of various inflammasomes types signaling by NLRP3 (Fig. 3G and H), AIM2, NLRP1 and NLRC4 (Fig. 5I and J), suggesting LND is a broad-spectrum anti-inflammatory agent. In addition to NLRP3 inflammasomes, there are amplifying evidences that other inflammasome types are involved in human disease. The AIM2 inflammasome is activated in ischemic mouse brains [65], and NLRC4 and NLRP3 activate an LPS-induced inflammasome in astrogliosis and microgliosis [66], while NLRP6 and NLRP3 inflammasomes negatively regulate NAFLD/NASH progression [67]. Therefore, LND may be effective in treating complex inflammatory diseases in which multiple inflammasomes are activated. Consistent with our data, antibody that specifically recognizes the CARD domain can inhibit ASC and inflammasome activation [68]. Additionally, an anti-ASC monoclonal antibody of IC-100 could suppress the immune-inflammatory response in EAE model [69]. Small molecular MM01 that interferes with ASC speck formation and ASC oligomerization inhibits inflammation in a mouse model of inflammasome-induced peritonitis [60].

Moreover, ASC oligomerization not only plays a key role in inflammasome assembly, the novel function of ASC specks has also been largely uncovered. Extracellular ASC specks can serve as danger signals to propagate inflammatory signals [17, 20]. Immune cells can take up extracellular ASC specks that maintain their ability to cause the maturation of pro-IL-1β. The deposition of ASC specks in tissues can not only induce strong neutrophil infiltration [17], but also exacerbate inflammation and Aβ deposition by forming complexes with Aβ extracellularly [18]. Besides, extracellular ASC can regulate adaptive immunity, and augmentation of ASC specks in lymph nodes increases the amplitude of T-cell differentiation, proliferation, and effector functions [19]. Furthermore, extracellular ASC specks have been detected in the serum of patients with chronic respiratory diseases and patients with autoinflammatory diseases [17, 20]. ASC proteins in the cerebrospinal fluid of patients with subarachnoid hemorrhage and TBI are potential biomarkers of disease severity, outcome, and secondary mechanisms of injury that impair recovery which can be used as adjuncts to clinical predictors [21]. Given the multiple functions of ASC in disease pathology mentioned above, the direct targeting ASC by LND imply its obvious advantages over other NLRP3 inhibitors.

Our data show that LND could inhibit the oligomerization of ASC and block the interaction between ASC and pro-casp-1 without affecting the binding of NLRP3 and ASC (Fig. 5), suggesting that LND may be directly bound with ASC. The direct interaction is verified by molecular simulation docking, SPR experiment and DARTS assay. Molecular mock docking identifies several amino acid residues involved in the binding of the CARD structural domain of ASC to LND including I155, F163, T166, W169, L178, L192 and S195 (Fig. 6A). W169G mutations almost completely disrupted ASC^CARD^ filament formation [58]. L178A and L192A mutations have been reported to produce a significant reduction in the number of ASC specks [60]. This suggests that LND may also bind to these amino acid residues to block the ASC oligomerization and ASC speck formation. The specific binding conformation and site of ASC and LND need to be further examined.

Hexokinase is one of key glycolytic enzymes that catalysis the first irreversible step, which has been demonstrated to involve in the regulation of inflammasome activation. Glycolysis is reported to be essential for NLRP3 activation, while the glycolytic inhibition may also act as an NLRP3 inflammasome inducer under distinct certain circumstances [70]. HK1-dependent glycolysis is reported to be required for NLRP3 but not AIM2 inflammasome activation, which is inhibited by HK1 shRNA and glycolytic inhibitor of 2-DG [71]. Another research identifies the subcellular localization of hexokinase also controls the bacterial peptidoglycan-activated NLRP3 inflammasome in macrophages [72]. Hexokinase competitive inhibitor of N-acetylglucosamine (NAG) is a degradation product of peptidoglycan (PGN) which is one of the components of the cell wall of Gram-positive bacteria. Stimulation of macrophages with NAG leads to the dissociation of hexokinase from the outer membrane of mitochondria which lead to the cytosol release of mitochondrial DNA to activate NLRP3 inflammasome [72]. Our results show that the mRNA (Additional file 1: Fig. S3A) and protein levels (Fig. 4A) of HK2 but not HK1 mRNA abundance (Additional file 1: Fig. S3A) increased during the activation of NLRP3 inflammasomes induced by LPS and ATP treatment in BMDM. However, the results using HK2 silencing (Fig. 4B and C) and HK2-knockout in macrophages (Fig. 4D and E) show that inhibiting HK2 did not block NLRP3 inflammasome activation. These different observations above may be related to cell types, kinds of stimuli, and stimulation intensities used in these studies, and the roles of glycolysis and glycolytic enzymes in inflammasome activation need to be further explored.

In conclusion, our study shows the remarkable therapeutic effects of LND on animal models of inflammasome-driven diseases, including EAE, acute ischemic stroke and LPS-induced sepsis. Moreover, LND strongly inhibits inflammasome activation via binding with ASC and preventing its oligomerization. This finding identifies LND as an inflammasome inhibitor via targeting ASC, suggesting the potential application of LND for inflammatory diseases and providing a leading compound for direct inhibitor of ASC.

## Supplementary Information

**Additional file 1:** **Fig S1.** LND attenuates the pathological process of EAE mice (A) EAE was induced in mice by MOG35~55 peptides and pertussis toxin as described in the Materials and Methods. Clinical scores of mice administered vehicle or LND daily after EAE onset; n = 10. (B) Weight change after EAE induction; n = 10. (C) Percentage of CD45+ gated on live cells and CD45+CD4+, CD45+CD8+ and CD45+CD11b+ cells gated on live cells from the mononuclear cell population in the CNS; the cells were isolated on day 15 after EAE induction. n = 5 The data are expressed as the mean ± SEM. *P < 0.05; **P < 0.01; ***P < 0.001; ****P < 0.0001; NS, not significant. Unpaired t test for A, C, D and E, and multiple unpaired t test for G. **Fig S2.** LND does not affect the priming stage of LPS induced inflammasome activation (A) Western blot analysis of mature IL-1β and cleaved caspase-1 (p20) in the supernatants (SN) of LPS-primed J774A.1 cells treated for 0.5 h with LND (0.2 mM) and then stimulated with ATP (5 mM) for 0.5 h. Western blot analysis of pro-IL-1β and pro-caspase-1 in the lysates of J774A.1 cells. (B and C) ELISA analysis of IL-1β (B) and TNF-α (C) in the supernatants of J774A.1 cells. (D) LDH levels in the supernatants of J774A.1 cells. (E-G) mRNA levels of IL-1β, IL-18 and TNF-α in J774A.1 cells that were pretreated with LND for 30 min and then stimulated with LPS for 3 h. (H) Western blot analysis of NLRP3, pro-IL-1β and pro-caspase-1 in lysates of J774A.1 cells that were pretreated with LND for 30 min and then stimulated with LPS for 3 h. The data are expressed as the mean and ± SEM. *P < 0.05; **P < 0.01; ***P < 0.001; ****P < 0.0001; NS, not significant. The data were analyzed by one-way ANOVA and Tukey’s test. **Fig S3.** Lonidamine inhibits NLRP3 inflammasome activation independent of HK2 in J774A.1 cells. (A) The mRNA levels of LND-targeted genes in LPS-primed BMDMs treated with LND for 30 min and then stimulated with ATP for 3 h were analyzed by qRT–PCR. (B) Western blot analysis of IL-1β and cleaved caspase-1 (p20) in the supernatants (SN) and pro-IL-1β, pro-caspase-1 and HK2 in the lysates of LPS-primed J774A.1 cells transfected with control, scrambled siRNA or HK2-specific siRNA as indicated for 24 h and then stimulated with ATP. (C) ELISA analysis of IL-1β in the supernatants of J774A.1 cells. (D) Western blot analysis of IL-1β and cleaved caspase-1 (p20) in the culture supernatants (SN) of LPS-primed BMDMs from HK2-/-mice that were then stimulated with ATP. Western blot analysis of pro-IL-1β, pro-caspase-1 and HK2 in the lysates of those cells. The data are expressed as the mean and ± SEM. *P < 0.05; **P < 0.01; ***P < 0.001; ****P < 0.0001; NS, not significant. The data were analyzed by one-way ANOVA and Tukey’s test. **Fig S4.** LND does not inhibit ROS production (A) The percentage of ROS+ cells among LPS-primed BMDMs treated with or without LND and then stimulated with ATP was examined by FACS. The data were analyzed by one-way ANOVA and Tukey’s test. The data are expressed as the mean and ± SEM. *P < 0.05; **P < 0.01; ***P < 0.001; ****P < 0.0001; NS, not significant. **Fig S5.** LND does not interact with NLRP3. Recombinant ASC and NLRP3 protein solution were coupled with CM5 chip (BR100530, Cytiva) to saturation, which was pre-balanced according to the manufacturer's instructions. LND injected into the flow cell at different concentrations with a flow rate of 5 μl/min at 25 °C to detect the response values. The response values obtained by injecting blank basic running buffer without LND was used as control. The kinetic parameters of the interaction and the affinity constants were calculated with Biacore T100 evaluation software. A to B, Sensorgrams of LND binding to the recombinant protein of ASC (A) or NLRP3 (B) obtained from surface plasmon resonance (SPR) analysis.

**Additional file 2.** The sequences of siRNA and primers.

## Data Availability

The datasets supporting the conclusions of this article are included within the article and its additional files.

## Abbreviations

ASC: Apoptosis-associated speck-like protein containing a CARD
LND: Lonidamine
MS: Multiple sclerosis
PRR: Pattern recognition receptor
NOD: Nucleotide-binding oligomerization domain
NLRP3: NOD-like receptor protein 3
AIM2: Absent in melanoma 2
AIM2: Melanoma 2 and pyrin
GSDMD: Gasdermin D
HK2: Hexokinase 2
MCAO: Transient middle cerebral artery occlusion
BMDMs: Murine bone marrow-derived macrophages
NEK7: NIMA-related kinase 7
MSU: Monosodium urate
SPR: Surface plasmon resonance
DARTS: Drug affinity responsive target stability
EAE: Experimental autoimmune encephalomyelitis
