# Supplementary material for: Directly targeting ASC by lonidamine alleviates inflammasome-driven diseases
Source: J Neuroinflammation. 2022 Dec 28;19:315. doi: 10.1186/s12974-022-02682-w (PMC9798610; doi:10.1186/s12974-022-02682-w)
Supplement: Supplementary file 1 — Additional file 1: Fig S1. LND attenuates the pathological process of EAE mice (A) EAE was induced in mice by MOG35~55 peptides and pertussis toxin as described in the Materials and Methods. Clinical scores of mice administered vehicle or LND daily after EAE onset; n = 10. (B) Weight change after EAE induction; n = 10. (C) Percentage of CD45+ gated on live cells and CD45+CD4+, CD45+CD8+ and CD45+CD11b+ cells gated on live cells from the mononuclear cell population in the CNS; the cells were isolated on day 15 after EAE induction. n = 5 The data are expressed as the mean ± SEM. *P < 0.05; **P < 0.01; ***P < 0.001; ****P < 0.0001; NS, not significant. Unpaired t test for A, C, D and E, and multiple unpaired t test for G. Fig S2. LND does not affect the priming stage of LPS induced inflammasome activation (A) Western blot analysis of mature IL-1β and cleaved caspase-1 (p20) in the supernatants (SN) of LPS-primed J774A.1 cells treated for 0.5 h with LND (0.2 mM) and then stimulated with ATP (5 mM) for 0.5 h. Western blot analysis of pro-IL-1β and pro-caspase-1 in the lysates of J774A.1 cells. (B and C) ELISA analysis of IL-1β (B) and TNF-α (C) in the supernatants of J774A.1 cells. (D) LDH levels in the supernatants of J774A.1 cells. (E-G) mRNA levels of IL-1β, IL-18 and TNF-α in J774A.1 cells that were pretreated with LND for 30 min and then stimulated with LPS for 3 h. (H) Western blot analysis of NLRP3, pro-IL-1β and pro-caspase-1 in lysates of J774A.1 cells that were pretreated with LND for 30 min and then stimulated with LPS for 3 h. The data are expressed as the mean and ± SEM. *P < 0.05; **P < 0.01; ***P < 0.001; ****P < 0.0001; NS, not significant. The data were analyzed by one-way ANOVA and Tukey’s test. Fig S3. Lonidamine inhibits NLRP3 inflammasome activation independent of HK2 in J774A.1 cells. (A) The mRNA levels of LND-targeted genes in LPS-primed BMDMs treated with LND for 30 min and then stimulated with ATP for 3 h were analyzed by qRT–PCR. (B) Western bl [file 12974_2022_2682_MOESM1_ESM.docx]

**Additional Figures**

**
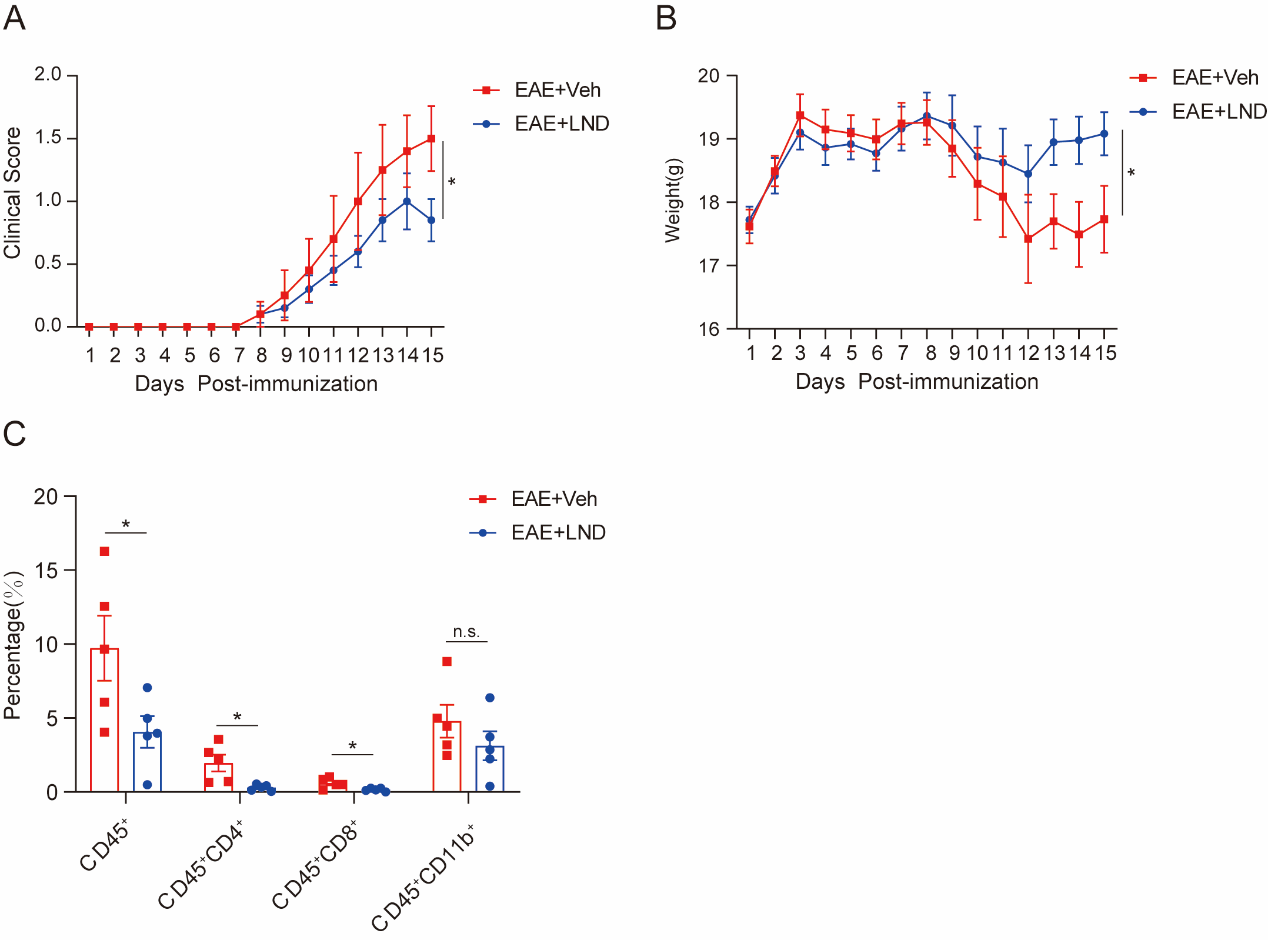
**

**Figure S1** **LND attenuates the pathological process of EAE mice (A)** EAE was induced in mice by MOG35~55 peptides and pertussis toxin as described in the Materials and Methods. Clinical scores of mice administered vehicle or LND daily after EAE onset; n = 10. **(B)** Weight change after EAE induction; n = 10. (C) Percentage of CD45^+^ gated on live cells and CD45^+^CD4^+^, CD45^+^CD8^+^ and CD45^+^CD11b^+^ cells gated on live cells from the mononuclear cell population in the CNS; the cells were isolated on day 15 after EAE induction. n = 5 The data are expressed as the mean ± SEM. *P < 0.05; **P < 0.01; ***P < 0.001; ****P < 0.0001; NS, not significant. Unpaired t test for A, C, D and E, and multiple unpaired t test for G.


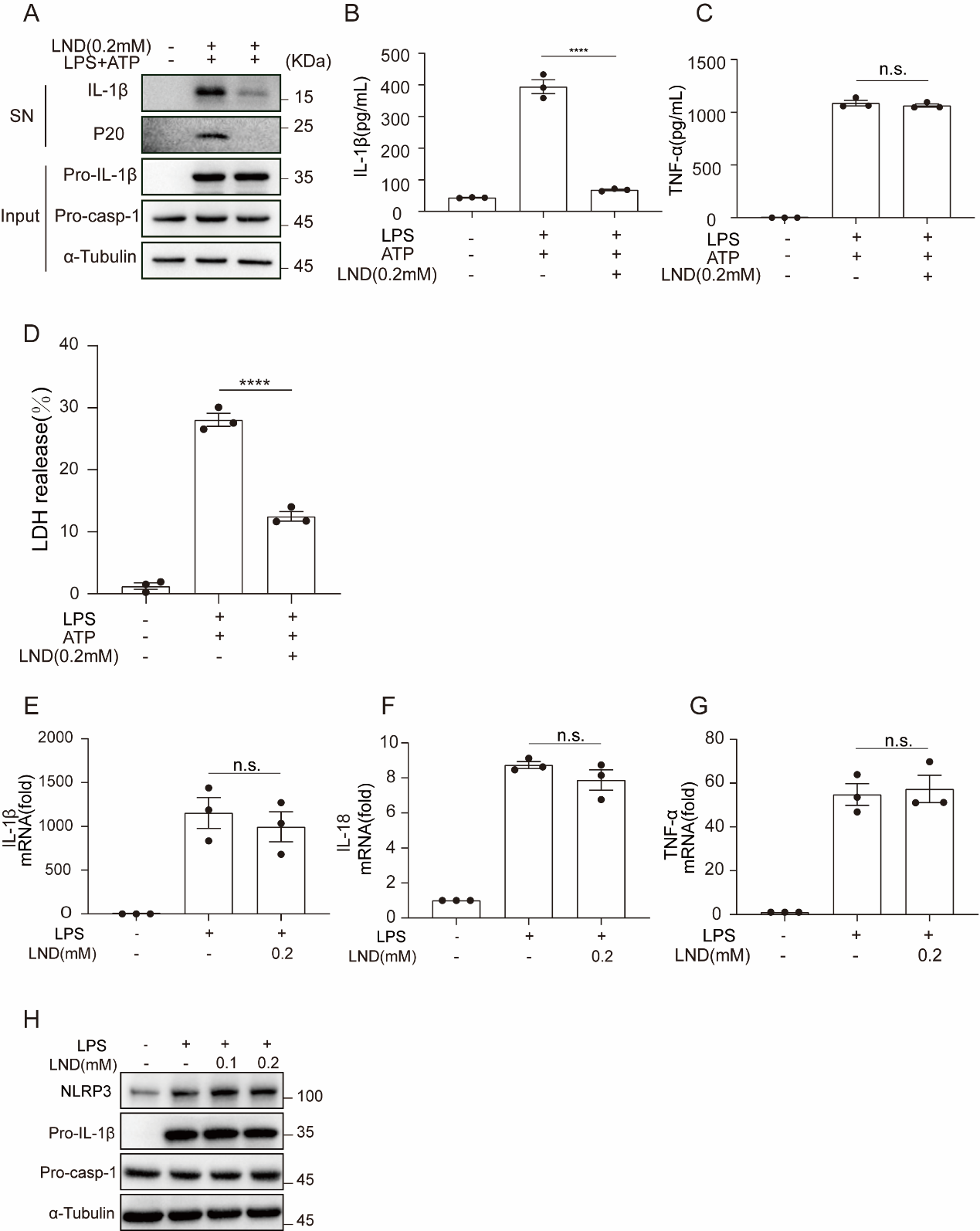


**Figure S2 LND does not affect the priming stage of LPS induced inflammasome activation (A)** Western blot analysis of mature IL-1β and cleaved caspase-1 (p20) in the supernatants (SN) of LPS-primed J774A.1 cells treated for 0.5 h with LND (0.2 mM) and then stimulated with ATP (5 mM) for 0.5 h. Western blot analysis of pro-IL-1β and pro-caspase-1 in the lysates of J774A.1 cells. **(B and C)** ELISA analysis of IL-1β **(B)** and TNF-α **(C)** in the supernatants of J774A.1 cells. **(D)** LDH levels in the supernatants of J774A.1 cells. **(E-G)** mRNA levels of IL-1β, IL-18 and TNF-α in J774A.1 cells that were pretreated with LND for 30 min and then stimulated with LPS for 3 h. **(H)** Western blot analysis of NLRP3, pro-IL-1β and pro-caspase-1 in lysates of J774A.1 cells that were pretreated with LND for 30 min and then stimulated with LPS for 3 h. The data are expressed as the mean and ± SEM. *P < 0.05; **P < 0.01; ***P < 0.001; ****P < 0.0001; NS, not significant. The data were analyzed by one-way ANOVA and Tukey’s test.


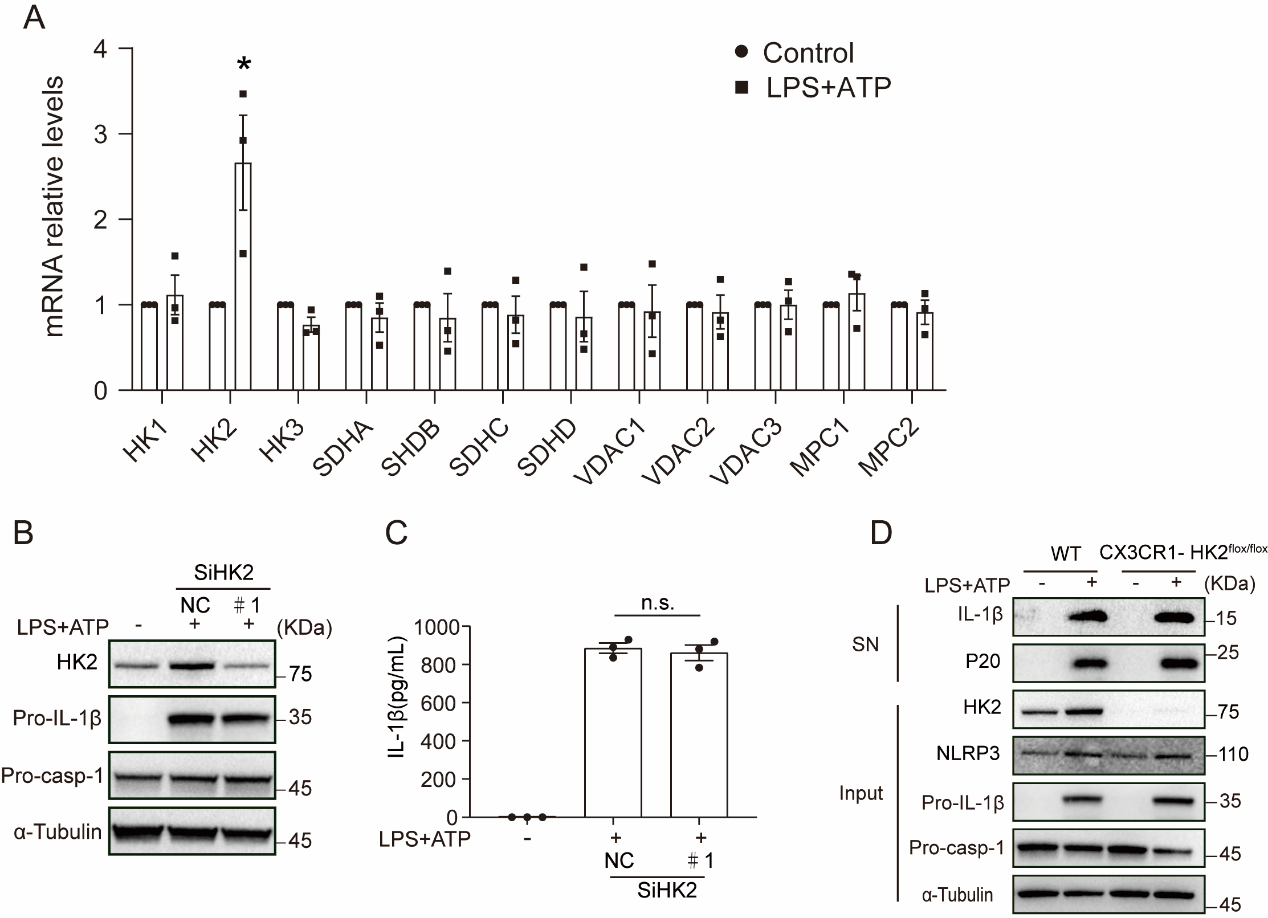


**Figure S3 Lonidamine inhibits NLRP3 inflammasome activation independent of HK2 in J774A.1 cells. (A)** The mRNA levels of LND-targeted genes in LPS-primed BMDMs treated with LND for 30 min and then stimulated with ATP for 3 h were analyzed by qRT–PCR. **(B)** Western blot analysis of IL-1β and cleaved caspase-1 (p20) in the supernatants (SN) and pro-IL-1β, pro-caspase-1 and HK2 in the lysates of LPS-primed J774A.1 cells transfected with control, scrambled siRNA or HK2-specific siRNA as indicated for 24 h and then stimulated with ATP. **(C)** ELISA analysis of IL-1β in the supernatants of J774A.1 cells. **(D)** Western blot analysis of IL-1β and cleaved caspase-1 (p20) in the culture supernatants (SN) of LPS-primed BMDMs from HK2^-/-^mice that were then stimulated with ATP. Western blot analysis of pro-IL-1β, pro-caspase-1 and HK2 in the lysates of those cells. The data are expressed as the mean and ± SEM. *P < 0.05; **P < 0.01; ***P < 0.001; ****P < 0.0001; NS, not significant. The data were analyzed by one-way ANOVA and Tukey’s test.


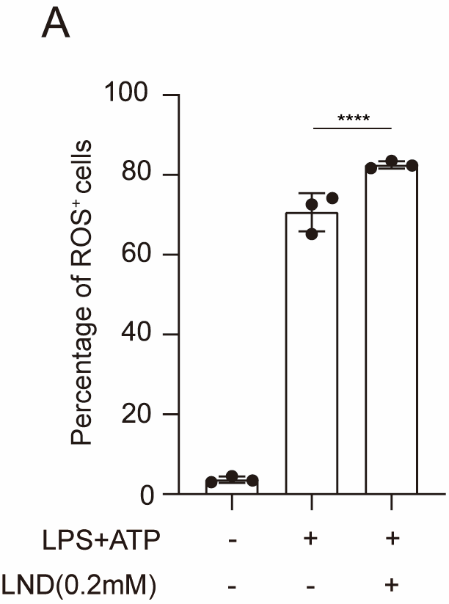


**Figure S4 LND does not inhibit ROS production (A)** The percentage of ROS+ cells among LPS-primed BMDMs treated with or without LND and then stimulated with ATP was examined by FACS. The data were analyzed by one-way ANOVA and Tukey’s test. The data are expressed as the mean and ± SEM. *P < 0.05; **P < 0.01; ***P < 0.001; ****P < 0.0001; NS, not significant.


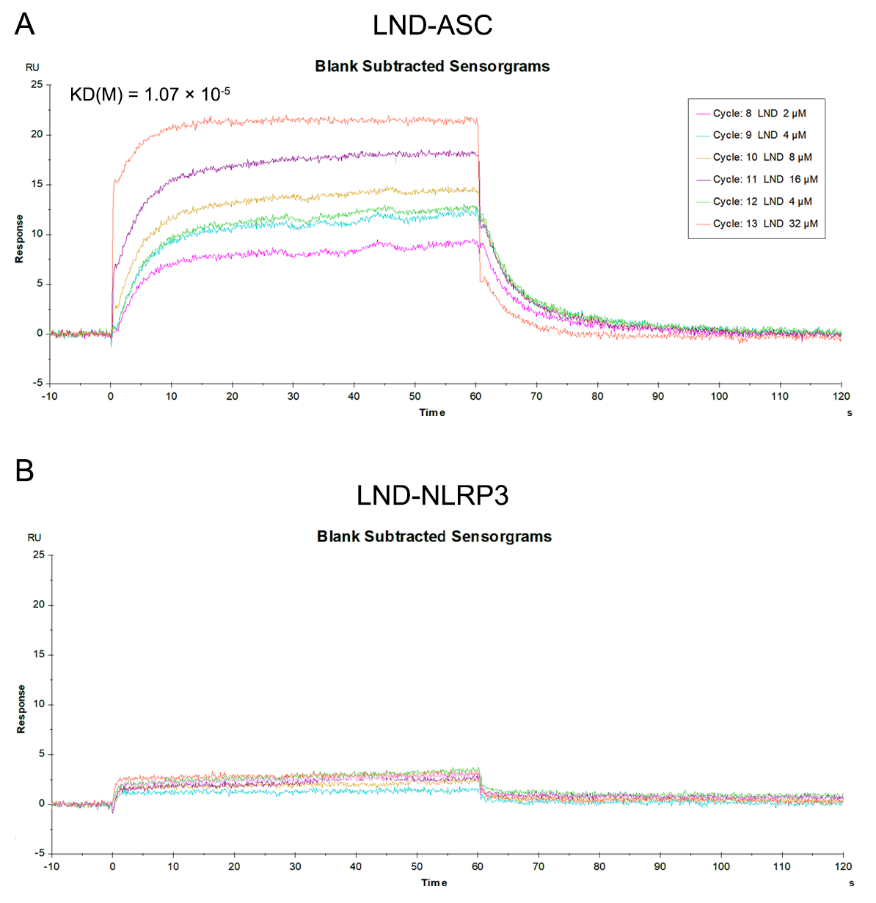


**Figure S5** **LND does not interact with NLRP3.** Recombinant ASC and NLRP3 protein solution were coupled with CM5 chip (BR100530, Cytiva) to saturation, which was pre-balanced according to the manufacturer's instructions. LND injected into the flow cell at different concentrations with a flow rate of 5 μl/min at 25 °C to detect the response values. The response values obtained by injecting blank basic running buffer without LND was used as control. The kinetic parameters of the interaction and the affinity constants were calculated with Biacore T100 evaluation software. **A to B**, Sensorgrams of LND binding to the recombinant protein of ASC **(A)** or NLRP3 **(B)** obtained from surface plasmon resonance (SPR) analysis.
