# Supplementary material for: Directly targeting ASC by lonidamine alleviates inflammasome-driven diseases
Source: J Neuroinflammation. 2022 Dec 28;19:315. doi: 10.1186/s12974-022-02682-w (PMC9798610; doi:10.1186/s12974-022-02682-w)
Supplement: Supplementary file 2 — Additional file 2. The sequences of siRNA and primers. [file 12974_2022_2682_MOESM2_ESM.docx]

**Real-time reverse transcription PCR**

The sequences of the primers are as follows:

HK1: CCCCACCGTCAACATGATCG, GAAATCCCCCTTTTCTGAGCC

HK2: CTGTTTCTGGAAACTTGAGGCCC, AGAGATACTGGTCAACCTTCTGC

HK3: CTGAATCTCCCAGGAATGCTT, TGCCTGTCAGTGTTACCCAC

SDHA: GAACACTCCAAAAACAGACCTGC, TCCACCACTGGGTATTGAGTAG

SDHB: ATTTACCGATGGGACCCAGAC, GTCCGCACTTATTCAGATCCAC

SDHC: CCCACCTGAATGCTCAGCTTT, AGAGGACGGTTTGAACTCGTG

SDHD: TGGTCAGACCCGCTTATGTG, GGTCCAGTGGAGAGATGCAG

VDAC1: GAGTATGGGCTGACGTTTACAG, GAGCTTCAGTCCACGAGCAAG

VDAC2: CACTGGTAAAGTTAGCGGGAC, CCAGAGTGTTATCGGTGTTCCA

VDAC3: GGGAAAGCATCAGGCAACCTA, GTCTGTATTCCACTTTTGGGTGA

MPC1: ATGAGTACGCACTTCTGGGG, CGCCCACTGATAATCTCTGGA

MPC2: AATTGAGGCCGCTTTACAACC, CACACACCAATCCCCATTTCA

IL-1β: TGCCACCTTTTGACAGTGATG, AAGGTCCACGGGAAAGACAC

IL-18: GACTCTTGCGTCAACTTCAAGG, CAGGCTGTCTTTTGTCAACGA

TNF-α: TCTGTCTACTGAACTTCGGGGTG, ACTTGGTGGTTTGCTACGACG

β-actin: CTAGGCACCAGGGTGTGATG, GTACATGGCTGGGGTGTTGA

**siRNA sequences**

The siRNA sequences are as follows:

siHK1-001: GCTGCTGAATAAAGCCATT

siHK1-002: GCTTATGAAGAACCTTCTT

siHK2-001: CCAAAGATGTCTCGGATAT

siHK2-002: GCAACATCCTGATCGATTT
